# Patient-generated health data: Impact on promoting patient-centered point of care tobacco treatment in patients with cancer

**DOI:** 10.1017/cts.2025.77

**Published:** 2025-04-28

**Authors:** Jessica Liu, Timothy B. Baker, Jingling Chen, Nina Smock, Nicholas B. Griffith, Ramaswamy Govindan, Paula Goldberg, Jodi Thole, Kristin Daly, James Reddy, Alex T. Ramsey, Laura J. Bierut, Aimee James, Robert A. Schnoll, Ross C. Brownson, Li-Shiun Chen

**Affiliations:** 1 Elson S Floyd College of Medicine, Washington State University, Spokane, WA, USA; 2 Department of Medicine, University of Wisconsin School of Medicine and Public Health, Madison, WI, USA; 3 Department of Psychiatry, Washington University School of Medicine, St. Louis, MO, USA; 4 Washington University School of Medicine in St. Louis, St. Louis, MO, USA; 5 Alvin J. Siteman Cancer Center at Barnes-Jewish Hospital and Washington University School of Medicine, St. Louis, MO, USA; 6 Department of Medicine, Washington University School of Medicine, St Louis, MO, USA; 7 Division of Public Health Sciences, Department of Surgery, Washington University School of Medicine, St. Louis, MO, USA; 8 Department of Psychiatry and Abramson Cancer Center, University of Pennsylvania, Philadelphia, PA, USA; 9 Prevention Research Center, Brown School at Washington University in St. Louis, St. Louis, MO, USA; 10 Department of Surgery, Division of Public Health Sciences, and Alvin J. Siteman Cancer CenterWashington University School of Medicine, St. Louis, MO, USA

**Keywords:** Patient-generated health data, health informatics, tobacco treatment, cancer prevention, translation

## Abstract

**Introduction:**

Guideline-based tobacco treatment is infrequently offered. Electronic health record-enabled patient-generated health data (PGHD) has the potential to increase patient treatment engagement and satisfaction.

**Methods:**

We evaluated outcomes of a strategy to enable PGHD in a medical oncology clinic from July 1, 2021 to December 31, 2022. Among 12,777 patients, 82.1% received a tobacco screener about use and interest in treatment as part of eCheck-in via the patient portal.

**Results:**

We attained a broad reach (82.1%) and moderate response rate (30.9%) for this low-burden PGHD strategy. Patients reporting current smoking (*n* = 240) expressed interest in smoking cessation medication (47.9%) and counseling (35.8%). As a result of patient requests via PGHD, most tobacco treatment requests by patients were addressed by their providers (40.6–80.3%). Among patients with active smoking, those who received/answered the screener (*n* = 309 ) were more likely to receive tobacco treatment compared with usual care patients who did not have the patient portal (*n* = 323) (OR = 2.72, 95% CI = 1.93–3.82, *P* < 0.0001) using propensity scores to adjust for the effect of age, sex, race, insurance, and comorbidity. Patients who received yet ignored the screener (*n* = 1024) compared with usual care were also more likely to receive tobacco treatment, but to a lesser extent (OR = 2.20, 95% CI = 1.68–2.86, *P* < 0.0001). We mapped observed and potential benefits to the Translational Science Benefits Model (TSBM).

**Discussion:**

PGHD via patient portal appears to be a feasible, acceptable, scalable, and cost-effective approach to promote patient-centered care and tobacco treatment in cancer patients. Importantly, the PGHD approach serves as a real world example of cancer prevention leveraging the TSBM.

## Introduction

Cancer patients are particularly vulnerable to the negative consequences that tobacco use has on treatments and mortality. Each puff of tobacco contains 60 well-established carcinogens that can lead to gene mutations that stop normal control of cellular growth and increase cancer risk [1]. Forty percent of all cancer diagnoses in the United States link back to tobacco use, including lung, stomach, kidney, pancreas, colon, and many others [2]. Nearly nine out of ten lung cancer deaths are caused by smoking or exposure to second-hand smoke [3]. Cancer patients who continue to use tobacco have an increased risk of second cancers [4] and decreased survival rates [5,6]. Despite this, many cancer patients continue to smoke or experience relapse after diagnosis. Paul and colleagues [7] found that 63% of cancer patients who smoked at the time of diagnosis continued to smoke post-diagnosis. Additionally, about 15% of those patients who quit smoking before cancer diagnosis experienced smoking relapse after cancer diagnosis. A cross-sectional analysis by Gritz and colleagues [8] concluded that 56% of cancer survivors who smoked at cancer diagnosis continued to smoke, with over half making unsuccessful attempts to quit in the last 12 months and 15% who quit experiencing relapse [8].

The American Society of Clinical Oncology states that stopping cigarette use after cancer diagnosis has benefits including longer and better quality of life, faster and more successful recovery from treatment, and lower risk of secondary cancers and infections (ASCO) [9,10]. The National Cancer Institute Monograph 23 notes that cancer patients who smoke face an increase in mortality due to heart disease, noncancer pulmonary disease, and stroke in addition to cancer [6]. Peppone and colleagues [11] found that patients who continue to smoke after a cancer diagnosis had significantly higher total symptom burden than nonsmoking patients at 6 months follow-up [11]. Because of the known benefits of smoking cessation, it is important that cancer patients have access to smoking cessation programs and resources [6,12].

The National Comprehensive Cancer Network (NCCN) guidelines recommend that anyone with cancer who smokes has a treatment plan that includes pharmacotherapy and behavioral therapy such as evidenced-based apps, text, and state Quitline, and close follow up [10]. The benefits of pharmacotherapy such as nicotine replacement and varenicline outweigh their risks, making them safe and effective for patients with cancer [10,13].

While smoking cessation treatments are effective, too few cancer patients receive such treatment [6]. These barriers include a lack of awareness of the post-diagnosis benefits of cessation, environmental stressors, addiction to smoking, fatalism, perceived lack of support and clear cessation messaging from health professionals [14,15]. Evidence suggests a misaligned perspective between patients and providers that patient interest in tobacco treatment is often under-estimated by the providers [16,17]. In practice, only 40% of providers actively assist their patients with quitting or refer them to treatment, creating a critical missed opportunity [18]. In fact, providers face multiple barriers to their offering and delivering smoking treatment: e.g., time constraints, lack of knowledge and skills, lack of practice support, and lack of accountability [19]. A low-barrier method for offering and supporting smoking cessation treatment is clearly needed.

To close this patient vs. provider perspective gap, traditional patient outreach strategies can be costly and have low reach [20,21]. EHR-enabled patient-generated health data (PGHD) hold promise for eliciting patient health concerns and increasing patient-centered treatment that is tailored to patient preferences and satisfaction with high reach and low cost.

Use of Patient-generated health data (PGHD) is growing and evolving to develop more patient-centered approaches to healthcare [22]. Further, PGHD can be helpful in tracking symptom management and improving health outcomes in oncology care [23]. PGHD and use of digital therapeutics are not replacements for care delivery, but instead should supplement clinician-supported care. EHR-enabled, low burden strategies have shown high reach and effectiveness in cancer clinics [24]. Rexhepi and colleagues [25] found that cancer patients are more likely than other patients to use online electronic health records demonstrating that electronic, low-burden approaches may be a useful way for patients to communicate healthcare needs in oncology settings.

PGHD can be a powerful tool for examining gaps in tobacco treatment. Using the electronic health record to elicit and transmit PGHD has the potential to significantly enhance delivery of patient-centered approaches to tobacco treatment.

There is a need to better document the impacts of patient-centered assessment strategy such as PGHD. One useful framework for mapping these impacts is the Translational Science Benefits Model (TSBM) [26]. The TSBM measures the impact of scientific discoveries beyond traditional metrics, including (1) clinical and medical benefits; (2) community and public health benefits; (3) economic benefits; and (4) policy and legislative benefits.

Using data from a QI initiative, this study evaluated the impact of PGHD on promoting patient-centered point of care tobacco treatment in patients with cancer. Specifically, we evaluate 1) the reach and patient response to PGHD regarding tobacco use and treatment interest, 2) whether PGHD promotes patient-centered care, 3) whether use of PGHD increases overall tobacco treatment, 4) potential disparities associated with EHR-enabled PGHD, and 5) the impacts of PGHD using the TSBM. We hypothesize that the PGHD intervention will be associated with increased tobacco treatment compared with patients not receiving the intervention. This approach, if effective, may be an example of research that can have broad impact beyond just traditional metrics like publications to include innovations in health-care delivery consistent with the Translational Science Benefits Model [26].

## Methods

### Study design

The goal of this quality improvement (QI) initiative was to increase patient-centered point of care tobacco treatment with an EHR-enabled PGHD outreach strategy. We implemented a tobacco screener as part of eCheck-in via a patient portal, MyChart, within a large medical oncology clinic in the Midwest region of the US that uses the electronic health record (EHR) system, Epic (Epic Systems, Verona, WI). The PGHD strategy is a systematic outreach effort to facilitate communications between patients and healthcare providers. In this study, we presented results from July 1, 2021 to December 31, 2022. The study was approved by the Institutional Review Board as a Quality Improvement (QI) project.

### PGHD tobacco screener workflow

**Figure 1.**
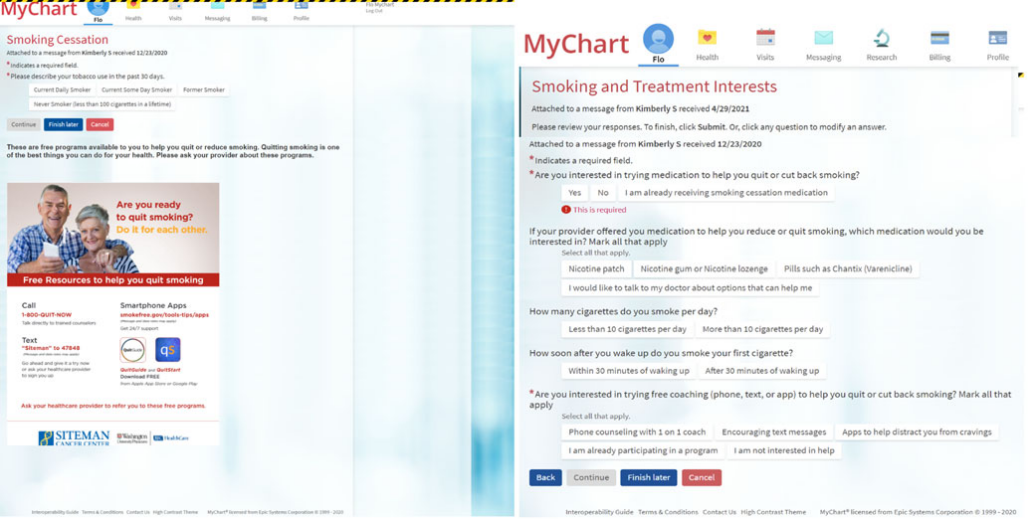
Patient-generated health data: pre-appointment tobacco screener during eCheck-in via the patient portal (MyChart) in electronic health record. The tobacco screener is delivered to outpatient return oncology visits every 90 days to ask patients about tobacco use, offer education via handout, and assess interest in smoking cessation treatments. Then, the clinical staff team assists in prescribing treatments and ordering counseling referral. The automatic system arranges for reassessment at next return visit 90 days later.

**Figure 2.**
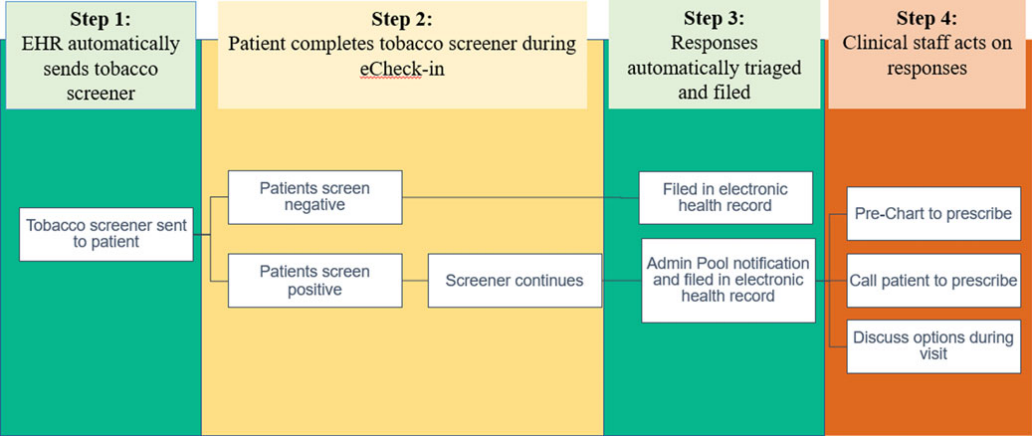
Patient-generated health data: Clinical workflows for patients and providers.

*Step 1: EHR automatically sends tobacco screener.* This QI initiative took place in an adult medical oncology clinic. The tobacco screener was sent to all patients within 7 days via MyChart prior to scheduled follow-up visits during eCheck-in. Patients can answer the screener within the 7 day period or when they arrive at the clinic. The tobacco screener was automatically suppressed with a response and repeated every 90 days thereafter if there was a follow up visit (Fig. 1, 2).

*Step 2: Patient receives and completes the tobacco screener during eCheck-in.* The tobacco screener assesses smoking status (Fig. 1). Free smoking education is provided via image of a flyer. The screener ends with patients selecting “Former Smoker” or “Never Smoker.” For patients selecting “Current Daily Smoker” or “Current Some Day Smoker,” the screener continues to assess treatment interest regarding medication or counseling. Patients were asked about their interest in pharmacotherapy (nicotine patch, gum, lozenge, varenicline, or discussing with the doctor) and counseling (phone counseling, text counseling, or app-based counseling).

*Step 3: Patient responses are automatically sent to providers’ InBasket for patient-centered decision-making.* Once the screen is submitted, the administration and leadership of the clinic receive an InBasket notification, a MyChart alert, about the respondents’ answers.

*Step 4: Provider teams can respond to patient responses regarding tobacco use and treatment interest.* Provider team nurse coordinators review InBasket patient responses and determine proper responses such as discussing tobacco treatment by phone or during the upcoming appointments (Fig. 2). Any treatment ordered pended by the nurses will be reviewed and approved by the physicians. This workflow allows providers to recognize patient interest, leading to more efficient patient-centered treatment. Providers receive feedback about every 6 months to encourage them to review and address patient-reported data.

### Data and outcomes

Data on the tobacco screener, patient encounter, and medication database were extracted from the EHR. The primary outcome was tobacco treatment, defined as patients receiving medication, brief advice, or offer of additional counseling such as phone-based, text-based, or app-based counseling. Specifically, the source of these outcome data is discrete fields from the EHR, designed in a smoking module as part of the electronic health record-enabled evidence-based tobacco treatment (ELEVATE) program described in prior research [27,28]. Medication is based on the prescription database and tobacco treatment is defined by FDA-approved medication (e.g., nicotine replacement, varenicline, bupropion prescribed as a smoking cessation aid). Brief advice is a checkbox for the provider team to advise patients with a verbal script “One of the best things you can do for health is to quit smoking.” Offer and referral of additional counseling options is a Best Practice Advisory for the provider team to do closed-loop referrals to these counseling services.

In addition, we evaluated factors that may be related to tobacco treatment including age, sex, race, health insurance coverage, and comorbidities. To minimize self-report biases, we used these strategies: 1) Training of medical assistants to ask about any smoking in the past 30 days. 2) Defining smoking cessation or former smoking when smoking occurred more than 30 days ago. 3) Allowing patients to self-report their smoking status via the screener in addition to answering the rooming staff about their smoking status.

### Statistical analysis

Descriptive statistics and Chi square tests were used to compare the patient characteristics across three patient groups. Group A includes patients who received/answered the screener. Group B includes patients who received/ignored the screener. Group C includes patients who did not have MyChart. Patients have multiple encounters during this timeframe. We defined current smoking if patients were documented as currently smoking in any of the encounters during this timeframe. As a result, analysis is done at the level of unique patients. Multivariable logistic regression models were used to compare tobacco treatment across three patient groups (patients who received/answered the screener vs. patients who received/ignored the screener vs. patients who did not have the patient portal). Covariates for the multivariable logistic regression models included age, sex, race, insurance status, and comorbidity.

We have used propensity score methods to evaluate the association between PGHD and tobacco treatment. Because these 3 patient groups were not randomized, there was selection bias such that the probability of receiving a strategy (e.g., using MyChart or responding to the tobacco screener in our study) was not equal across groups. We used Inverse probability of treatment weighting (IPTW), a method that uses propensity scores to adjust for confounding variables in observational studies. Propensity scores are the probability of receiving a strategy, given an individual characteristic. IPTW involves weighting individuals by the inverse of their propensity score to create a synthetic sample where treatment assignment is independent of measured covariates. Covariates used to calculate the propensity scores include age, sex, race, insurance, and comorbidity. Next, we checked the balancing ability of IPTW. Then we evaluated the effect of a strategy while using these weights and generate confidence intervals while taking the weighing into account.

Analyses were done using SAS 9.4. The primary hypothesis is to evaluate whether PGHD increases tobacco treatment. We conducted three tests to compare three patient groups (patients responding to the screener, patients receiving but not responding to screener, and patients not having the patient portal) with three paired comparisons. The secondary hypothesis is to evaluate whether patient characteristics differ across the three patient groups. We conducted five tests to evaluate five patient characteristics (age, sex, race, insurance, and comorbidity). The tertiary hypothesis is to evaluate whether the effect of PGHD differs by race. We conducted one test to evaluate whether there is a significant interaction between race and PGHD on the outcome of receiving tobacco treatment. Overall, we have three primary tests, five secondary tests, and one tertiary test. We have adjusted the significance threshold for type I error alpha value from 0.05 to 0.005, given the number of statistical tests.

## Results

### PGHD implementation via patient portal, MyChart

Among a total of 12,777 patients, 82.1% (*n* = 10,496) patients had access to the patient portal, MyChart, and received a tobacco screener as part of eCheck-in via MyChart about tobacco use and interest in treatments. A small group of patients who received the tobacco screener via MyChart (*n* = 336) were missing encounter data and not included in the analyses of tobacco treatment across patient groups (Table 1).

**Table 1.** Sample characteristics

Overall (*N* = 12,777)*			Patients responding to screener (*n* = 3243)		Patients receiving but not responding to screener (*n* = 7253)*		Patients not having the patient portal (*n* = 2617)		*A vs C*	*B vs C*	*A vs B*
**Sex**	*n*	*%*	*n*	*%*	*n*	*%*	*n*	*%*	*p*	*p*	*p*
Female	7045	55.2	1813	55.9	3682	53.2	1550	59.2	0.0105	<0.0001	0.0117
Male	5732	44.8	1430	44.1	3235	46.8	1067	40.8			
**Race**			*n*	%	*n*	%	*n*	%			
White	10,101	79.1	2860	88.2	5154	74.5	2087	79.8	<0.0001	<0.0001	<0.0001
Black/African American	2155	16.9	284	8.8	1522	22.0	349	13.3			
Other	521	4.0	99	3.1	241	3.5	181	6.9			
**Age (years)**											
18–40	1311	10.3	330	10.2	738	10.7	243	9.3	<0.0001	0.0711	0.0001
41–54	2327	18.2	677	20.9	1203	17.4	447	17.1			
55–64	3412	26.7	883	27.2	1852	26.8	677	25.9			
>64	5727	44.8	1353	41.7	3124	45.2	1250	47.8			
**Insurance**											
Medicare	6050	47.4	1442	44.5	3394	49.1	1214	46.4			
Private	5685	44.5	1634	50.4	2891	41.8	1160	44.3	<0.0001	0.0571	<0.0001
All others	1042	8.1	167	5.2	632	9.1	243	9.3			
Comorbidities*											
0–1	8588	67.3	1990	61.4	4568	66.0	2030	77.6	<0.0001	<0.0001	<0.0001
2+	4189	32.7	1253	38.6	2349	34.0	587	22.4			

These data were extracted from the electronic health record for 12,777 mutually exclusive patients from July 2021–December 2022. Of the patients with MyChart, A & B (*n* = 10,496), a small group of patients whom received the tobacco screener via MyChart and did not respond *(*n* = 336) were missing encounter data and not included in this analysis. Using chi-square tests, A vs C and A v B comparison shows patients who were Female, White, over the age of 64, had private health insurance and had less comorbidities were more likely than males to receive treatment. In the B vs C comparison, patients who were female, white, and had less comorbidities were more likely to receive treatment. The total *N* is A + B + C minus 336 that were missing encounter data due to missed appointment and not included in this analysis. Other race includes American Indian or Alaska Native, Asian, Other Pacific Islander, all other racial, and missing (1.45%) backgrounds. Other insurance includes Medicaid, other state insurance, Tricare, all others, and missing (0.42%).

### Technology-enabled PGHD showed a broad reach among oncology patients

**Figure 3.**
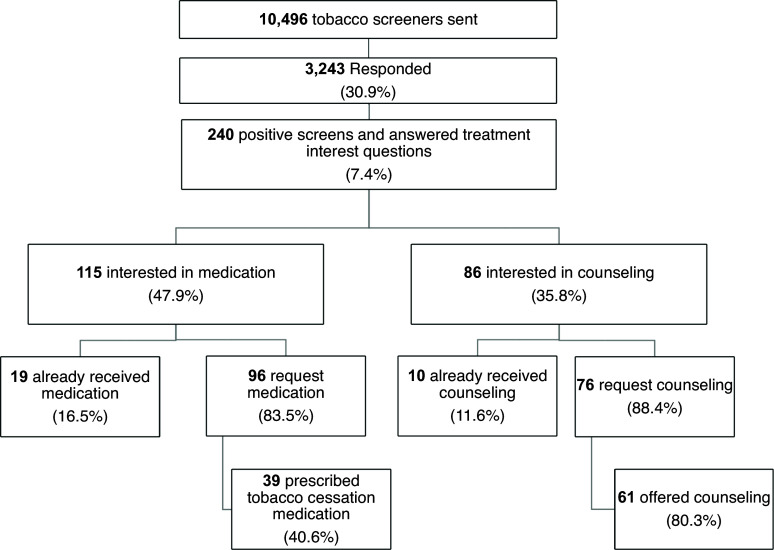
Patient-generated health data: Patients share treatment interest and receive tobacco treatment. response rate: Of the 10,496 screeners sent, 7,253 did not respond (69.1%) and 3,243 responded (30.9%). Tobacco use status: 1093 reported former smoking, 1906 never smoking, 156 current daily smoking, and 88 current someday smoking. Of 244 who screened positive for tobacco use (some day or daily smoking status), 4 did not answer treatment interest question. Interest in treatment: 240 answered treatment interest question. Of the 115 patients interested in medication, 19 were already receiving medication and 96 are newly interested. Of the 86 interested in counseling, 10 were already receiving counseling and 76 are newly interested.

Patient-centered outreach via PGHD has a broad reach. We found a moderate patient response rate (30.9%) for the tobacco screener during pre-appointment eCheck-in (3243 of 10,496, Fig. 3). In this self-selected patient group who received and responded to the screener, self-reported smoking status was as follows, 156 (4.8%) current daily smoking, 88 (2.7%) current someday smoking, 1093 (33.7%) former smoking, 1906 (58.8%) never smoking (Fig. 3).

### Many patients who smoke expressed interest in tobacco treatments

A substantial percentage of patients with active smoking expressed interest in smoking cessation medication (115 of 240, 47.9%) and counseling (86 of 240, 35.8%) (Fig. 3). Interest in medication includes interest in nicotine patch (40, 41.7%), nicotine gum/nicotine lozenge (28, 29.2%), varenicline (24, 25.0%), or wanting to discuss medications with their physician (38, 39.6%). Interest in counseling includes phone-based counseling (15, 17.4%), text-based counseling (41, 47.7%), and app-based counseling (35, 40.7%).

### Many patients expressing treatment interest did receive treatment via PGHD

Providers addressed most patient requests made via PGHD. Among 115 patients interested in medication, 19 (16.5%) were interested in medication and already receiving medication based on their electronic medical record data. Among 96 patients who reported interest and had not already received medication, 40.6% of them received tobacco treatment medication within the next year of their requests (Fig. 3).

Among 86 patients interested in counseling, 10 (11.6%) were interested in counseling and already receiving counseling based on the electronic medical record. Among 76 patients who reported interest but had not received counseling, 61 (80.3%) were offered counseling within the next year of their requests (Fig. 3).

### Implementation of PGHD and prevalence of tobacco treatment among all clinic patients

Table 1 shows the characteristics of patients in three groups. Group A includes patients who received/answered the screener (*n* = 3243). Group B includes patients who received/ignored the screener (*n* = 7253). Group C includes the usual care patients who did not have the patient portal MyChart (*n* = 2617).

To evaluate the impact of PGHD on receipt of tobacco treatment, we identified patients who smoke in these three patient groups using EHR data. We evaluated the impact of PGHD on tobacco treatment in 3 patient groups as shown in Table S1. Group A includes patients who actively smoked and received/answered the screener (*n* = 309). Group B includes patients who actively smoked and received/ignored the screener (*n* = 1024). Group C includes the usual care patients who actively smoked and did not have the patient portal MyChart (*n* = 323).

**Figure 4.**
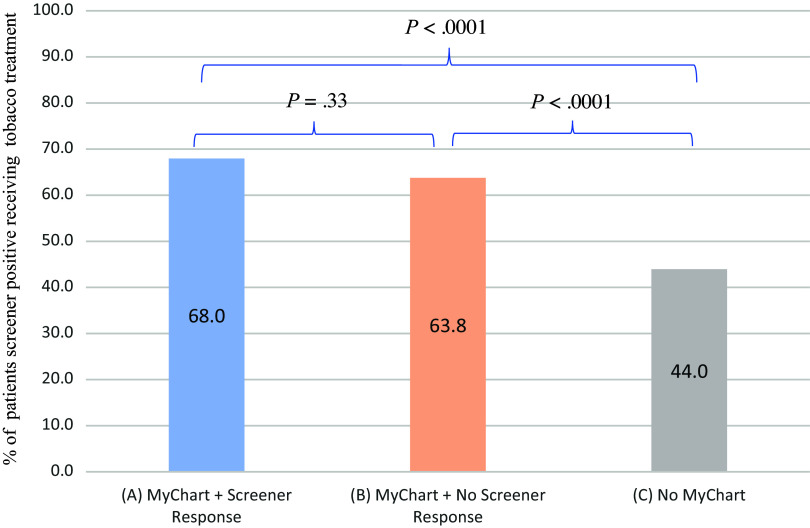
Tobacco treatment in three patient groups: Patients responding to screener, patients receiving/not responding to screener, and patients without the patient portal. (1) Patients responding to screener vs. Patients not having the patient portal are associated with more treatment received in multivariate logistic regression (OR, 2.72, 95%; CI, (1.94–3.82); *P* < 0.0001), adjusted for age, sex, race, insurance status, and comorbidity. (2) Patients receiving by not responding to screener vs. Patients not having the patient portal are associated with more treatment received in multivariate logistic regression (OR, 2.20, 95%; CI, (1.68–2.86); *P* < 0.0001), adjusted for age, sex, race, insurance status, and comorbidity.(3) Patients responding to screener vs. Patients receiving but not responding to screener are associated with more treatment received in multivariate logistic regression (OR, 1.16, 95%; CI, 0.86–1.58); *P* = 0.33), adjusted for age, sex, race, insurance status, and comorbidity. (4) *N* = 309 (group A, patients who smoked and responded to screener), *N* = 1024 (group B, patients who smoked and did not respond to screener), *N* = 323 (group C, patients who did not have the portal).

Using the propensity score methods, we found significant associations between patient groups and receipt of tobacco treatment. Covariates used to calculate the propensity scores include age, sex, race, insurance, and comorbidity. Compared to patients not having the portal, patients responding to the screener and patients not responding to the screener were associated with higher tobacco treatment (OR = 2.72, 95 % CI 1.94–3.82, *P* < 0.0001; OR = 2.20, 95 % CI 1.68–2.86, *P* < 0.0001) as shown in Table 2 and Fig. 4. The standardized differences are shown in Table S2.

**Table 2.** Association of patient-generated health data use patterns and likelihood of tobacco treatment

	OR (95% CI)	*P* Value
**Intervention Groups**		
Patients not having the patient portal	Reference	
Patients responding to screener	2.72 (1.94–3.82)	< 0.0001
Patients receiving, but not responding to screener	2.20 (1.68–2.86)	< 0.0001

1. We have used propensity score methods to evaluate the association of PGHD and tobacco treatment. Because these 3 patient groups were not randomized, there was selection bias such that the probability of receiving a strategy (e.g., using MyChart or responding to the tobacco screener in our study) was not equal across groups. We used Inverse probability of treatment weighting (IPTW), a method that uses propensity scores to adjust for confounding variables in observational studies. Propensity scores are the probability of receiving a strategy, given an individual characteristics. IPTW involves weighting individuals by the inverse of their propensity score to create a synthetic sample where treatment assignment is independent of measured covariates. Next, we checked the balancing ability of IPTW. Then we evaluated the effect of a strategy while using these weights and generate confidence intervals while taking the weighing into account.

2. 1656 tobacco users, *N* = 309 (group A, patients who smoked and responded to screener), *N* = 1024 (group B, patients who smoked and did not respond to screener), *N* = 323 (group C, patients who did not have the portal).

In addition, we used multivariable regressions as an alternative approach and reached similar results. Patients responding to the screener and who smoke were more likely to receive tobacco treatment (medication or counseling) compared with usual care patients who did not have the patient portal ) (OR = 2.65, 95 % CI = 1.90–3.70, *P* < .0001) adjusting for age, sex, race, insurance, and comorbidity. Similarly, patients who received yet ignored the screener, compared with usual care, were also more likely to receive tobacco treatment to a lesser extent (OR = 1.96, 95 %CI = 1.51–2.55), *P* < 0.0001) as shown in Table S3. We have conducted additional analyses with refined categories for covariates (race, ethnicity, insurance) and reached similar results.

In addition, we compared the two groups with MyChart: patients responding vs. not responding to PGHD. Using the propensity score methods, there was no significant difference between patients responding or not responding to screener and receipt of tobacco treatment (OR = 1.16, 95 % CI 0.86–1.58, *P =* 0.33). Covariates used to calculate the propensity scores include age, sex, race, insurance, and comorbidity. The standardized differences are shown in Table S4. Additionally, we used multivariable regression models and reached similar results. Patients who received/answered the screener) were more likely to receive tobacco treatment (medication or counseling) compared with patients who received/ignored the screener) (OR = 1.33, 95% CI = 1.01–1.76, *P =* 0.046) adjusting for age, sex, race, insurance, and comorbidity (Fig. 4, Table S5). Detailed information regarding tobacco treatment is in Table S1.

In addition, quit rates were the highest among patients responding to the screener (28.8%), then patients not responding to the screener (18.8%), then patients without the portal (18.0%). Among patients responding to the screener, quit rates were higher in those receiving tobacco treatment (31.0%) than those not receiving tobacco treatment (24.2%).

*6. Disparity in access and response to EHR-enabled PGHD, but not in the association of PGHD and receipt of tobacco treatment*

We compared characteristics across the 3 patient groups: A) patients who received/answered the screener, B) patients who received/ignored the screener, and C) patients who did not have the patient portal MyChart (Table 1). Patients who received/answered the screener were more likely to be White, younger, have private insurance and more comorbidities compared to the other groups. Building on the multivariable logistic regression model presented in Table 2, we tested the interaction (PGHD * race). Despite the difference in racial compositions across patient groups, we found that the effect of PGHD on tobacco treatment did not differ by race as indicated by no significant interaction between group and race (χ2 = 3.36, df = 4, *P* = 0.50).

## Discussion

This study shows that an innovative EHR-enabled PGHD is a low-burden strategy with a broad reach with moderate patient response as part of cancer care. Further, many patients were interested in tobacco treatment and received such treatment as a result of their requests. As such, PGHD may be a promising implementation strategy to increase the offer and delivery of tobacco treatment. The PGHD strategy allows providers to have a more informative assessment of the patient’s tobacco use and treatment interest. Moreover, it may enhance patient awareness, education, and knowledge, thereby enabling successful patient-centered tobacco treatment during an oncology appointment.

These findings extend existing research on patient outreach. Before EHR-enabled PGHD, we piloted a traditional non-sustainable outreach with a nurse calling patients to discuss tobacco treatment and found a response rate similar to PGHD (reaching 4 out of 11 patients, 36%). Leveraging health informatics, not only is this EHR-enabled PGHD strategy cost effective using an automatic process, but also it is scalable. This PGHD strategy reached > 10,000 patients with a similar response rate and little provider time was required. Patient interest in treatment via the portal prompts providers to discuss or offer the desired tobacco treatment before, during, or after the clinic visit by removing barriers such as time constraints and improving accountability to discuss smoking cessation. Importantly, our findings showed an encouraging response rate (31%) compared with prior studies on use of MyChart [29]. Our response rate is comparable to existing studies that collect patient-generated data. Prior research suggests low rates (ranging from 13% to 42%) of responses despite the high reach of these automatic screeners [15,29,30]. Reasons why patients may skip the screener may include lack of time to complete the screener, thinking it may be irrelevant to their situation, and unwillingness to share their smoking status. eCheck-in completion rates have wide variation across clinics, populations, and time.

We found an increase in overall tobacco treatment in the patient group completing or receiving PGHD compared with those without the patient portal. Given that patients self-selected into these groups without randomization, multiple mechanisms could account for the observed patterns of outcomes. For instance, differences in patients who opted to receive patient portal messages might have differed from other patients in myriad ways that were difficult to control. Also, the point of care (POC)/PGHD approach entailed not only solicitation of responses from patients but also offered patient educational options, which might have yielded benefits that did not depend upon clinicians’ responses to PGHD information. Further, the POC/PGHD approach entailed some education for healthcare workers and this might have increased smoking treatment offers and support. Although mechanisms are unclear, there is evidence that screeners can be helpful in changing clinical behavior in other contexts as well, including diabetes control and adolescent health behaviors [31,32].

Some evidence supports that depending on the EHR/PGHD approach used, increasing the reach of smoking treatment offered overall, resulted in uneven reach across certain populations. Those who both received the messages and responded to them were more likely to be White, have private health insurance, and have greater numbers of comorbidities than those who did not both respond and receive the messages. We identified the disparity in patient access to EHR-enabled PGHD and the important needs to reduce the disparity with advancement of health informatics. Although there was no racial disparity in overall receipt of tobacco treatment or the effect of PGHD. The major disparity lies in the access and use of EHR-enabled PGHD. There may also be implicit bias among providers against patients without MyChart, leading to a lower likelihood of receiving treatment. A critical parallel effort is needed to enable PGHD for patients without access to the patient portal or expansion of patient portal access to all patients.

These results need to be considered with several limitations. First, smoking status is self-reported, voluntary, and un-verified. The population that engaged with PGHD may be more health-conscious and/or under-reporting smoking. This can explain why PGHD data showed a smoking prevalence of 7.5%, which is lower than the smoking rate documented in all medical oncology encounters of 13% (as assessed by medical assistants during rooming). Typically, these smoking status responses are documented in the EHR when the patient is being roomed. However, the eCheck-in process intentionally asks the patient to self-report their smoking status. This allows the patients to share their treatment needs without provider observation or questioning. It is possible that some patients may still under-report for concerns of social norms. Second, patients who are not fluent in English or have low literacy may have difficulty with the questionnaire or be deterred from completing it. Further, using a digital screener likely limited the user group to those who had access to technology. Another limitation of all patient portal-based intervention, including this intervention, is its limited reach among patients who do not have the patient portal or patients who do not complete pre-appointment eCheck-in. Future research is critically needed for strategies to collect patient-generated health data that are easily accessible for all patients and computability with the electronic health record (EHR). Third, some patient treatment requests were not addressed. Our QI team provided training and data feedback to providers in order to promote provider responses to patient requests via PGHD. Patient request regarding tobacco treatment may not be addressed given many competing health care needs and staff turnover in a busy oncology clinic. Fourth, this QI effort is not an experimental design, allowing conclusions about the effectiveness of PGHD without a randomization or quasi-experimental design. Because these patient groups were not randomized, there was selection bias such that the probability of receiving a strategy (e.g., using MyChart or responding to the tobacco screener in our study) was not equal across groups. To mitigate this problem, we have used propensity scores to adjust for confounding variables with the probability of receiving a strategy, given an individual characteristic. However, we acknowledge that these methods may only mitigate but not eliminate these negative effects. Further, EHR data is limited in documentation of smoking status or treatment delivery. These results were also limited by the use of discrete data in the EHR without analyzing texts in clinician notes. Future research is needed to control for many other factors (e.g., how comorbidities are assessed).

Given the low burden, high patient reach, positive satisfaction, and clinical impact of this PGHD strategy, we have not only continued this strategy in the large medical oncology clinic but also scaled this strategy to another medical oncology clinic. Rigorous evaluation of this approach for a longer duration and multiple clinic types can provide knowledge on its impact across populations and settings. Perhaps the most critical challenge is to increase the offer and reach of tobacco treatment to those with lower healthcare and technology engagement for cancer prevention. Future research is needed to enhance patient engagement by tailoring the educational information for patient characteristics.

**Figure 5.**
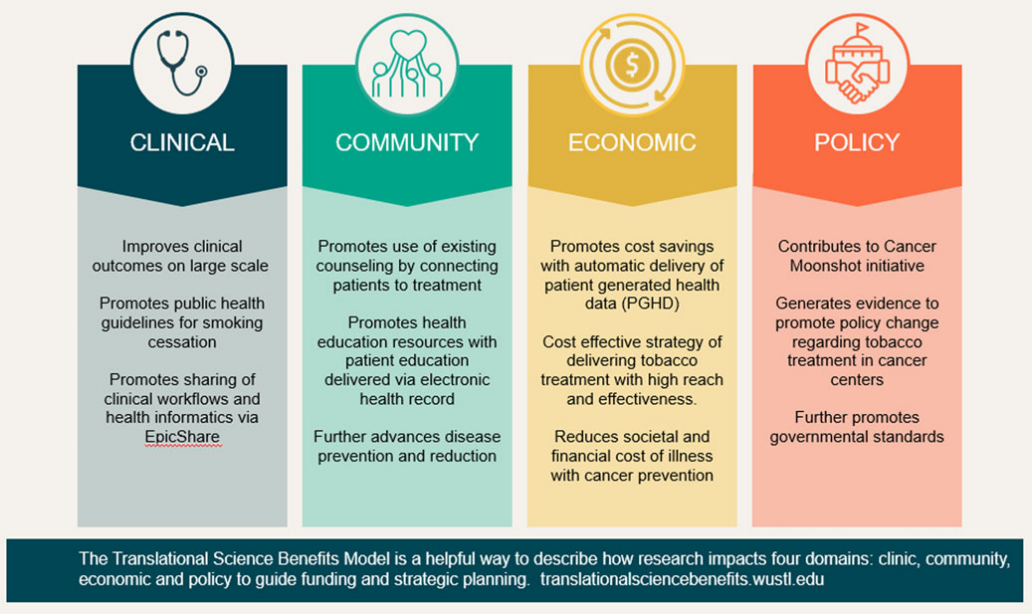
Using the Translational Science Benefits Model to document patient-centered tobacco treatment.

This evidence suggests that PGHD via patient portal is a feasible and acceptable approach to promote patient-centered care and tobacco treatment in cancer patients. Leveraging health informatics, this EHR-enabled PGHD strategy may involve low cost by using an automatic process to enhance its sustainability and scalability. Future research is needed to evaluate the cost effectiveness of this strategy along with other PGHD and implementation strategies. Importantly, the PGHD approach serves as a real world example of cancer prevention leveraging the Translational Science Benefits Model (TSBM) (Fig. 5). The impacts we have noted cut across multiple domains. At the clinical level, with potential to increase tobacco treatment prescriptions, both medically and through counseling, PGHD can improve clinical outcomes in these cancer patients and supports public health guidelines for smoking cessation. At the community level, PGHD also promotes the usage of existing health education and counseling in the community, supporting businesses of similar values. At the economic level, PGHD may be a low-cost, high-reach strategy to encourage smoking cessation for both patients and providers. At the policy level, PGHD promotes tobacco-reducing cancer prevention and supports current governmental standards to fight cancer.

In summary, PGHD is a low-burden, patient-centered strategy to promote tobacco treatment in medical oncology patients, with the goal of enhancing cancer outcomes, quality of life, and survival rates. This study lays the foundation for a future path of a digital transformation of equitable tobacco treatment for all patients.

## Supporting information

Liu et al. supplementary material Liu et al. supplementary material

## Supplementary material

The supplementary material for this article can be found at https://doi.org/10.1017/cts.2025.77
