# Supplementary material for: Patient-generated health data: Impact on promoting patient-centered point of care tobacco treatment in patients with cancer
Source: J Clin Transl Sci. 2025 Apr 28;9(1):e114. doi: 10.1017/cts.2025.77 (PMC12171923; doi:10.1017/cts.2025.77)
Supplement: Liu et al. supplementary material [file S2059866125000779sup001.docx]

Table S1: Tobacco Use and Treatment Across Patient Groups

| **Overall (N=12777)** | | | **Patients responding to screener (n=3243)** | | **Patients receiving but not responding to screener (n=7253)**** | | **Patients not having the patient portal (n=2617)** | |
| --- | --- | --- | --- | --- | --- | --- | --- | --- |
| **Tobacco Use** | *n* | *%* | *n* | *%* | *n* | *%* | *n* | *%* |
| Assessment | 12575 | 98.4 | 3239 | 99.9 | 6862 | 99.2 | 2474 | 94.5 |
| Smoking* | 1656 | 13.0 | 309 | 9.5 | 1024 | 14.9 | 323 | 13.1 |
| Medication | 470 | 28.4 | 99 | 32.0 | 297 | 29.0 | 74 | 22.9 |
| Advise | 678 | 40.9 | 149 | 48.2 | 458 | 44.7 | 71 | 22.0 |
| Behavioral Support Offered | 1235 | 74.6 | 231 | 74.8 | 803 | 78.4 | 201 | 62.2 |
| Behavioral Support Referred | 104 | 6.3 | 17 | 5.5 | 74 | 7.2 | 13 | 4.0 |
| Any Treatment | 1005 | 60.7 | 210 | 68.0 | 653 | 63.8 | 142 | 44.0 |

*smoking status documented in the electronic health record, higher than that reported in PGHD via MyChart.

**(n=336) were missing encounter data and not included in this analysis

Advice is brief advice given, Offer is offering additional counseling, Referral is referral to additional counseling, any treatment is advise, referral or medication

Of the patients with MyChart, patient portal (n=10,496), a small group of patients whom received the tobacco screener via MyChart (n=336) were missing encounter data and not included in this analysis.

Table S2. Propensity score diagnostics for all participants

|  | **A vs. C** | | **B vs. C** | |
| --- | --- | --- | --- | --- |
|  | **STDDIFF** | | **STDDIFF** | |
|  | **Before PS** | **After PS** | **Before PS** | **After PS** |
| Age | -0.18 | 0.013 | -0.064 | 0.010 |
| Sex | -0.00070 | -0.00078 | 0.14 | 0.027 |
| Race | -0.26 | -0.011 | 0.14 | 0.0084 |
| Insurance | -0.14 | -0.015 | -0.020 | -0.0023 |
| Comorbidity | 0.26 | -0.0029 | 0.34 | 0.013 |

N=309 (group A, patients who smoked and responded to screener), N=1024 (group B, patients who smoked and did not respond to screener), N=323 (group C, patients who did not have the portal)

PS: Propensity Score. STDDIFF: standardized difference

Table S3: Association of patient generated health data (PGHD) use patterns and likelihood of tobacco treatment

|  | OR (95% CI) | *P* Value |
| --- | --- | --- |
| **Sex** |  |  |
| Female | Reference |  |
| Male | 0.89 (0.73 - 1.10) | 0.27 |
| **Race** |  |  |
| White | Reference |  |
| Black or African American | 1.67 (1.31 - 2.12) | <.0001 |
| Other | 0.83 (0.43 - 1.60) | 0.57 |
| **Age (years)** |  |  |
| 18-40 | Reference |  |
| 41-54 | 1.06 (0.71 - 1.60) | 0.77 |
| 55-64 | 0.93 (0.63 - 1.37) | 0.71 |
| >64 | 0.95 (0.62 - 1.45) | 0.79 |
| **Insurance** |  |  |
| Private | Reference |  |
| Medicare | 1.20 (0.92 - 1.57) | 0.18 |
| All others | 1.23 (0.91 - 1.65) | 0.18 |
| **Comorbidities** |  |  |
| 0-1 | Reference |  |
| 2+ | 1.57 (1.26 - 1.96) | <.0001 |
| **Intervention Groups** |  |  |
| Patients not having the patient portal | Reference |  |
| Patients responding to screener | 2.65 (1.90 - 3.70) | <.0001 |
| Patients receiving, but not responding to screener | 1.96 (1.51 - 2.55) | <.0001 |

1656 tobacco users

1. Patients responding to screener (vs Patients not having patient portal) are associated with more treatment received in multivariable logistic regression (OR=2.65, 95% CI=1.90-3.70, *P*< .0001), adjusted for age, sex, race, insurance status, and comorbidity.
2. Patients receiving but not responding to screener (vs Patients not having patient portal) are associated with more treatment received in multivariate logistic regression (OR, 1.96 , 95% CI, (1.51-2.55); *P*< 0.0001), adjusted for age, sex, race, insurance status, and comorbidity.
3. Other race includes American Indian or Alaska Native, Asian, Other Pacific Islander, all other racial, and missing (1.45%) backgrounds. Other insurance includes Medicaid, other state insurance, Tricare, all others, and missing (0.42%)
4. N=309 (group A, patients who smoked and responded to screener), N=1024 (group B, patients who smoked and did not respond to screener), N=323 (group C, patients who did not have the portal)

Table S4. Propensity score diagnostics for all participants

|  | **A vs. B** | |
| --- | --- | --- |
|  | **STDDIFF** | |
|  | **Before PS** | **After PS** |
| Age | -0.012 | -0.030 |
| Sex | -0.14 | -0.0099 |
| Race | -0.43 | 0.053 |
| Insurance | -0.11 | -0.013 |
| Comorbidity | -0.084 | -0.0074 |

N=309 (group A, patients who smoked and responded to screener), N=1024 (group B, patients who smoked and did not respond to screener).

PS: Propensity Score. STDDIFF: standardized difference

Table S5: Tobacco Treatment and Patients responding vs. not responding to PGHD

|  | OR (95% CI) | *P* Value |
| --- | --- | --- |
| **Sex** |  |  |
| Female | Reference |  |
| Male | 0.89 (0.71 - 1.13) | 0.34 |
| **Race** |  |  |
| White | Reference |  |
| Black or African American | 1.60 (1.23 - 2.10) | 0.0005 |
| Other | 0.54 (0.23 - 1.27) | 0.16 |
| **Age (years)** |  |  |
| 18-40 | Reference |  |
| 41-54 | 1.20 (0.77 - 1.88) | 0.43 |
| 55-64 | 0.99 (0.65 - 1.53) | 0.98 |
| >64 | 1.00 (0.62 - 1.61) | 1.00 |
| **Insurance** |  |  |
| Private | Reference |  |
| Medicare | 1.17 (0.86 - 1.60) | 0.31 |
| All others | 1.04 (0.75 - 1.45) | 0.81 |
| **Comorbidities** |  |  |
| 0-1 | Reference |  |
| 2+ | 1.80 (1.41 - 2.31) | <.0001 |
| **Intervention Groups** |  |  |
| Patients receiving, but not responding to  screener | Reference |  |
| Patients responding to screener | 1.33 (1.01 - 1.76) | 0.0464 |

Patients Responding to Screener vs Patients Receiving, but Not Responding to Screener are associated with more treatment received in multivariate logistic regression (OR, 1.33 , 95% ; CI, (1.01-1.76); *P*=0.0464), adjusted for age, sex, race, insurance status, and comorbidity.

N=309 (group A, patients who smoked and responded to screener), N=1024 (group B, patients who smoked and did not respond to screener)
